# Bosutinib mitigates inflammation in experimental sepsis

**DOI:** 10.1111/eci.70055

**Published:** 2025-04-28

**Authors:** C. M. C. Cunha, V. H. P. Abreu, V. Estato, G. M. V. Soares, B. P. T. Moraes, G. P. Oliveira, J. D. Silva, P. L. Silva, R. Immler, P. R. Rocco, M. Sperandio, A. R. Silva, P. T. Bozza, H. C. Castro‐Faria‐Neto, C. F. Gonçalves‐de‐Albuquerque

**Affiliations:** ^1^ Laboratório de Imunofarmacologia Instituto Oswaldo Cruz, FIOCRUZ Rio de Janeiro Brazil; ^2^ Laboratório de Imunofarmacologia Instituto Biomédico, Universidade Federal Do Estado Do Rio de Janeiro Rio de Janeiro Brazil; ^3^ Laboratory of Pulmonary Investigation Carlos Chagas Filho Institute of Biophysics, Federal University of Rio de Janeiro Rio de Janeiro Brazil; ^4^ Walter Brendel Centre, Department of Cardiovascular Physiology and Pathophysiology Klinikum der Universität, Ludwig Maximilians University München Munich Germany

**Keywords:** bosutinib, neuroinflammation, sepsis, Src family tyrosine kinase inhibitors

## Abstract

**Background:**

Sepsis, a leading cause of death globally, lacks targeted and effective treatment. Its pathophysiology involves unbalanced inflammation, marked by a high release of inflammatory mediators, leukocyte recruitment, vascular changes and dysfunction of the nervous and respiratory systems. Src family tyrosine kinases (SFK) play a critical role in immune responses, and their inhibition can modulate excessive inflammation. This study investigates the potential of bosutinib, an SFK inhibitor, as a treatment for sepsis.

**Methods:**

Clinical signs, survival rates, systemic and neuronal inflammatory responses, cell recruitment, lung function and cerebral microcirculation were analysed in mice treated with bosutinib (3 mg/kg) or DMSO/saline followed by cecal ligation and puncture (CLP)‐induced sepsis.

**Results:**

Bosutinib treatment reduced the severity of sepsis, improved survival rates and reduced the levels of pro‐inflammatory cytokines and chemokines in peritoneal lavage, plasma and brain tissue. It also reduced cellular infiltration and bacterial growth at the infection site and protected lung function by reducing diffuse alveolar damage. Using intravital microscopy and laser speckle techniques, bosutinib improved capillary density and blood perfusion and reduced leukocyte recruitment and adhesion in the cerebral microcirculation of septic animals.

**Conclusions:**

Bosutinib pretreatment attenuated dysregulated inflammatory responses and neurovascular changes in experimental sepsis.

## Graphical abstract

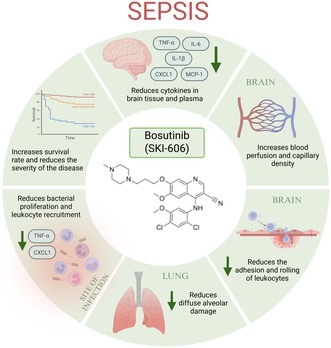

## 1 INTRODUCTION

Sepsis is a potentially fatal condition caused by a dysregulated host response to infection.^1^ Management relies on antibiotic therapy and control of clinical symptoms.^2^ Recognized by the World Health Organization as a global health priority, sepsis is a leading cause of mortality and morbidity in intensive care units.^3^ In 2017, sepsis affected an estimated 48.9 million people worldwide, resulting in 11 million deaths.^4^

Sepsis is characterized by sustained systemic inflammation and organ injury, often affecting areas distant from the primary focus of infection.^5^ The brain is notably impacted,^6^ leading to sepsis‐associated encephalopathy (SEA) in about 70% of patients. This condition involves glial hyperactivation, blood perfusion impairment and vascular and neuronal damage.^7^ Moreover, acute respiratory distress syndrome (ARDS) is a severe sepsis complication that significantly increases the mortality rate by causing lung inflammation, endothelial cell dysfunction and diffuse alveolar damage.^8^

Systemic inflammation in sepsis compromises endothelial cell function across different organs, leading to increased capillary permeability, impaired microcirculation, hypoxia, tissue injury and organ failure.^9^ Recruited neutrophils release tumour necrosis factor (TNF)‐α, interleukin (IL)‐1β, reactive oxygen species (ROS) and proteases, further activating the endothelium and exacerbating the host's inflammatory response.^10^

The Src family kinases (SFK) are nonreceptor protein tyrosine kinases that regulate immune responses, including chemotaxis, cell adhesion, ROS generation, cytokine secretion and endothelial permeability.^11^ In endothelial cells, SFK mediate neutrophil adhesion and increase vascular permeability.^12^ SFK are also involved in TLR‐activated pathways that produce inflammatory proteins during immune responses.^13^ Dysregulated SFK activity is linked to pathological events,^14^ and its inhibition can attenuate exaggerated leukocyte and endothelial responses in inflammation.^15^, ^16^

Originally developed for cancer treatment, SFK inhibitors (SFKIs) have shown immunomodulatory effects in inflammatory and autoimmune disorders.^17^ Bosutinib, a multiple kinase inhibitor used to treat chronic myeloid leukaemia, has demonstrated the ability to elevate IL‐10 levels, decrease IL‐6 and TNF‐α levels, and induce a resolving profile in LPS‐stimulated primary macrophages.^18^ Furthermore, bosutinib modulates systemic inflammation and mitigates neuroinflammation in mice subjected to an Alzheimer's disease model.^19^ Controlling excessive inflammatory cascades while restoring immune response balance is a promising therapeutic strategy in sepsis.^20^, ^21^ We hypothesize that bosutinib may modulate the exacerbated immune response in sepsis. For this purpose, this study evaluates the effect of bosutinib on clinical aspects, survival rate, bacterial growth, lung function, inflammatory parameters, cerebral microcirculation and brain blood perfusion in septic mice.

## 2 MATERIALS AND METHODS

### 2.1 Animals

Male Swiss‐Webster mice (27 g) from the Institute of Science and Technology of the Oswaldo Cruz Foundation, Rio de Janeiro, Brazil, were housed in ventilated racks (Alesco®, Brazil), equipped with a micro‐environmental ventilation system with a 12‐h light regime, a temperature of 21 ± 2°C, and humidity 55 ± 5%. All experimental procedures were carried out under the approval of the Ethics Committee on the Use of Animals of the Oswaldo Cruz Foundation (licence number L015/2015) and the Ethics Committee on Experimentation of the Federal University of the State of Rio de Janeiro (licence number 2019/03). All procedures were made according to Animal Research: Reporting of In Vivo Experiments (ARRIVE) guidelines.

### 2.2 Bosutinib treatment

**FIGURE 1:**
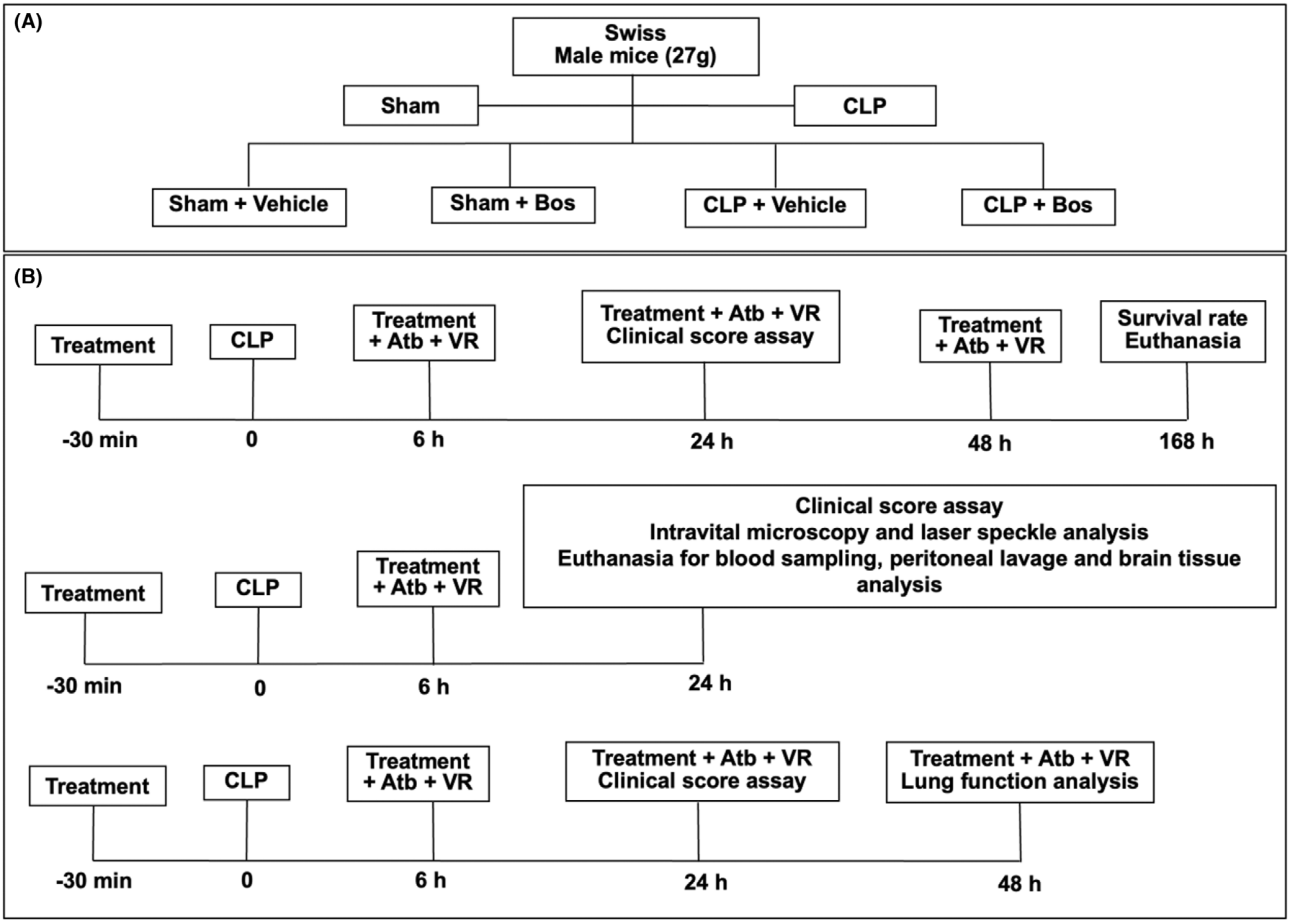
Upper panel: (A) Experimental group division. Swiss mice underwent cecal ligation and puncture (CLP) with sham animals as controls. Bos—Animals treated with bosutinib (3 mg/kg) before CLP. Vehicle—animals treated with vehicle (Saline and DMSO). Lower panel: (B) Timeline of analyses performed in the experimental design. Treatment—bosutinib or vehicle, Atb–meropenem antibiotic (10 mg/kg), VR, volume reposition (saline + glucose 20%).

Swiss mice received bosutinib (Cayman Chemical) (3 mg/kg) orally by gavage with a proper needle, in a volume of 100 μL for each animal, 30 min before and 6 h after sepsis induction. Control animals received saline/DMSO (vehicle) in the same volume as bosutinib treatment (Figure 1A).

### 2.3 Coecal ligation and puncture model

The experimental design is shown in Figure 1A. Briefly, animals received bosutinib or vehicle intraperitoneally (i.p.) 30 min before coecal ligation and puncture (CLP). The CLP model was conducted as recently described by Gonçalves‐de‐Albuquerque et al., 2018.^22^ All animals received antibiotic treatment with meropenem (10 mg/kg) and volume reposition (saline + glucose 20%) via i.p. route at 6 h for all experiments performed at 24 h after CLP, as follows: clinical score evaluation, intravital microscopy, laser speckle analysis, blood perfusion and sample collection (blood, peritoneal lavage and brain tissue). For lung function, analysis was performed within 48 h, so the animals received a second meropenem dose and volume reposition at 24 h. Finally, survival assessment was conducted at 144 h post‐CLP, and the animals received a third meropenem dose and volume reposition at 48 h (Figure 1B).

### 2.4 Assessment of sepsis severity

Twenty‐four hours after the CLP, physical and behavioural parameters, such as piloerection, altered breathing, change in stools, tearing, lack of exploration, impaired locomotion, contracted abdomen, absence of strength to grasp (weakness), altered body temperature, alertness and water turgor loss, were observed. Each observed sign added a point to the clinical score (0–11 points). Infection intensity was scored as follows: 0 (no clinical change), 1–3 (mild sepsis), 4–7 (moderate sepsis) and 8–11 (severe sepsis).

### 2.5 Anaesthesia

The animals were anaesthetised with ketamine (100 mg/kg) and xylazine (10 mg/kg) before CLP, laser speckle or intravital microscopy. In other experiments, Swiss mice were anaesthetised with isoflurane 24 h after CLP for perfusion and peritoneal lavage. In additional experiments, 48 h after sepsis induction, Swiss mice treated with bosutinib (3 mg/kg) or DMSO/saline were sedated [diazepam (1 mg/kg, ip)] and anaesthetised [ketamine (67 mg/kg, ip) and xylazine (30 mg/kg, ip)] for lung function analysis.

### 2.6 Mouse perfusion

For protein quantification in cerebral tissue, mice were perfused postmortem. To do this, the rib cage was opened via an incision. Thereafter, a 21‐gauge needle connected to a normal saline bag was inserted into the heart's left ventricle, and perfusion started. After perfusion, the skull was opened, and the brain was extracted. Brains perfused with sterile saline and EDTA were used for protein quantification and enzyme‐linked immunosorbent assay (ELISA) processing. The biological samples were stored at −80°C until ELISA processing.

### 2.7 Peritoneal lavage

The peritoneal cavity was washed with a cold, sterile saline solution (3 mL). Aliquots of the peritoneal lavages were used for total cell count and bacterial growth analysis (colony‐forming units (CFU) count). Red blood cells were lysed in Turk's solution (2% acetic acid), and the total cell count was performed in a Neubauer chamber (Neubauer Improved). Differential leukocyte count was done in cytocentrifuge smears stained with Panotic (Laborclin). The supernatant was collected by centrifugation and stored at −20°C to quantify cytokines. Aliquots of the peritoneal washes were plated on Difco soybean triptych agar (TSA) (BD) and incubated aerobically at 37°C for 24 h for CFU counting.

### 2.8 Lung function

Forty‐eight hours after sepsis induction, Swiss mice treated with bosutinib (3 mg/kg) or DMSO/saline were anaesthetised, tracheotomised, paralysed (vecuronium bromide, .005 mg/kg, iv) and ventilated with a constant flow ventilator (Samay VR15; Universidad de la Republica, Montevideo, Uruguay) with the following parameters: respiratory frequency of 100 breaths/min, tidal volume (VT) of .2 mL and fraction of inspired oxygen of .21. The anterior chest wall was surgically removed, and a positive end‐expiratory pressure (PEEP) of 2 cmH_2_O was applied to avoid alveolar collapse. Airflow and tracheal pressure (Ptr) were measured. In an open chest preparation, Ptr reflects transpulmonary pressure (PL). After a 10‐min ventilation period, static lung elastance (Est‚L) was calculated using the end‐inflation occlusion method.^23^ Data were analysed using ANADAT data analysis software (RHT‐InfoData, Inc., Montreal, Quebec, Canada). All experiments lasted less than 15 min.^24^

### 2.9 Protein quantification

Brains were extracted for ELISA in the sample buffer. Homogenized tissue was centrifuged, and the supernatant was collected. Total proteins in the supernatant were measured using the bicinchoninic acid (BCA) colourimetric method (Thermo Fisher Scientific). These data normalized the concentration of inflammatory mediators measured by ELISA.

### 2.10 Mediators of inflammation measurement

Levels of CXCL1, MCP‐1, TNF‐α, IL‐1β, IL‐6, VEGF, TGF‐β and IL‐10 in the supernatant of brain tissue, and CXCL1 and TNF‐α in the peritoneal fluid and plasma were quantified by ELISA according to the manufacturer's instructions (R&D Systems DuoSet).

### 2.11 Laser speckle

Briefly, the animals were anaesthetised, tracheostomized and mechanically ventilated (Ugo Basile) with room air. Blood pressure and heart rate were monitored via a right carotid artery connected to a Biopac Systems data acquisition system. After fixation in a stereotaxic structure, a cranial window was created by drilling the left parietal bone of the skull (1–5 mm laterally, between the coronal and lambdoid sutures) after exposing the dura mater and arachnoid membranes. The cranial window was filled with artificial cerebrospinal fluid (in mmol: NaCl, 132; CXCL1l 2.95, CaCl_2_ 1.71; MgCl_2_ .64; NaHCO_3_ 24.6; dextrose 3.71; urea 6.7 at 37°C and pH 7.35–7.45). Blood perfusion was monitored using a laser speckle contrast imaging system (LSCI) (Perimed), providing a blood perfusion index proportional to red blood cell concentration and average speed. The cranial window was positioned under LSCI light (785 nm wavelength), with body temperature maintained at 36°C (Harvard Apparatus). The distance between the laser source and the cranial window was 10 cm, according to the manufacturer's instructions. To assess microvascular cerebral blood flow in real‐time, initially, a region of standard interest (RSI) was defined, which is the position of the window opening in the skull, and the same RSI was used for all animals. Laser speckle images per second and the relative cerebral blood flow of all animals were analysed using the Perisoft software (Perimed) and expressed as an arbitrary perfusion unit (AP).

### 2.12 Intravital microscopy

Following laser speckle measurement, mice were placed under a vertical fixed‐stage microscope (Zeiss, Axio Scope) equipped with an x20/NA*xy* water immersion objective and an LED lamp coupled to a camera system (Zeiss Axiocam) for recording and subsequent processing using ZEN software (Zeiss). The procedure was done as follows.^25^ Briefly, the caudal vein was punctured for the administration of fluorescent markers. Fluorescein isothiocyanate (FITC‐150) labelled dextran (.1 mL) was administered intravenously, and the cerebral microvasculature was visualized. The fluorescent dye rhodamine 6G (.3 mg/kg) was administered intravenously to mark circulating leukocytes. Four venular segments were selected randomly (30–100 mm in diameter) and observed for 30 s in each preparation to analyse the leukocyte‐endothelium interaction. Rolling leukocytes were defined and counted as the number of cells crossing the given venular segment for 1 min. Adherent leukocytes were determined as the total number of leukocytes firmly adhered to the endothelium that did not move during 1 min of observation.

The density of functional capillaries, classified as the total number of spontaneously perfused capillaries (vessels with diameters less than 10 μm) per square meter of surface area (mm^2^), was determined by capillary branch count in random microscopic fields for 4 min.

### 2.13 Statistical analysis

Data were presented as mean ± standard error (SEM) and analysed using one‐way ANOVA followed by Newman–Keuls post‐hoc test using GraphPad Prism 5.0. Survival curves and comparisons between the curves were evaluated using the Mantel‐Cox log‐rank test. In both tests, values of *p* < .05 were considered significant.

## 3 RESULTS

### 3.1 Bosutinib improves sepsis survival and severity after coecal ligation and puncture (CLP)

**FIGURE 2:**
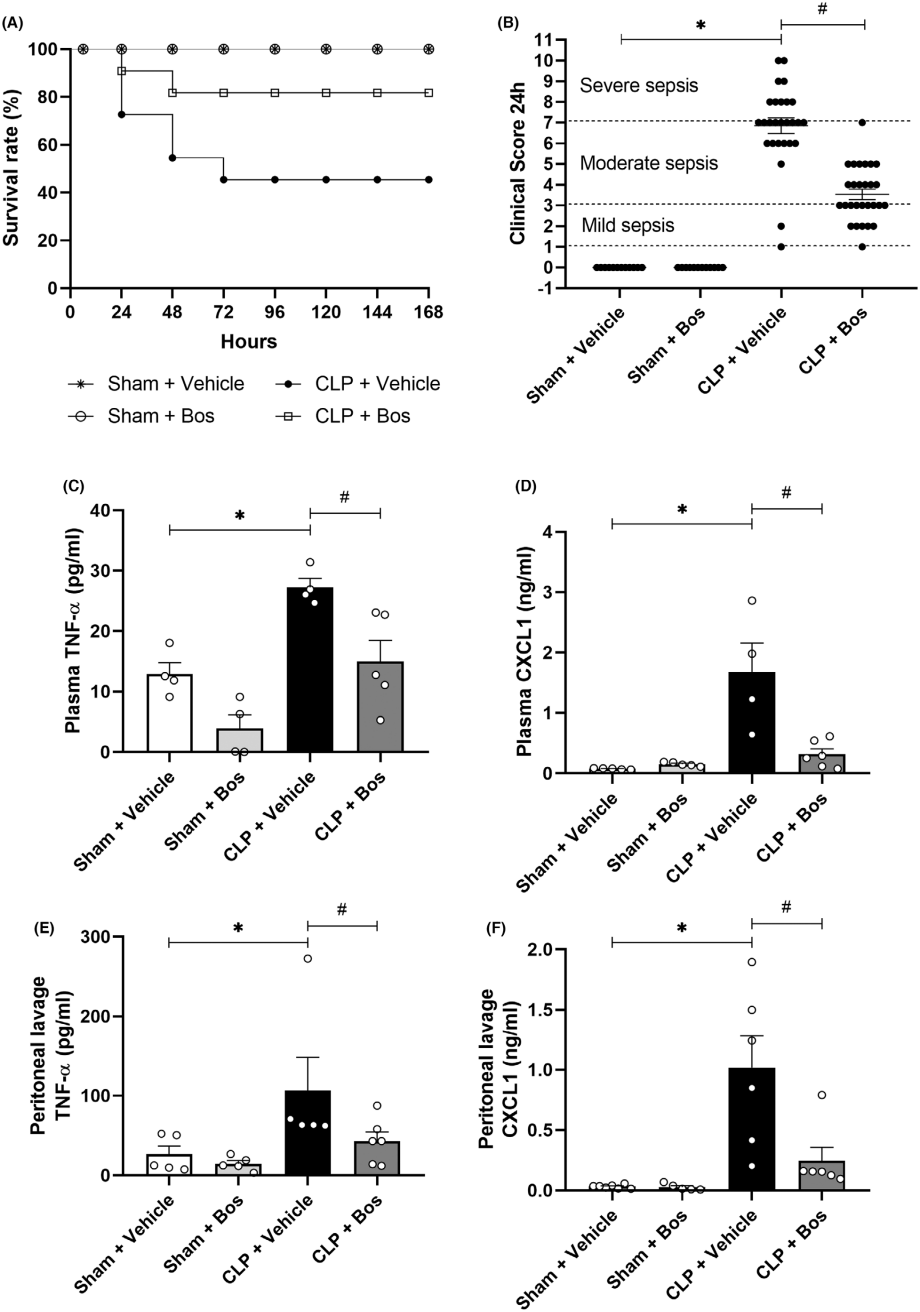
Effect of bosutinib on sepsis survival and severity. Swiss mice underwent cecal ligation and puncture (CLP). Sham animals were used as controls. Data are presented as mean ± SEM. (A) Quantification of the survival rate of sham and septic mice, treated or not with bosutinib, over 7 days (168 h). (B) The clinical score of the mice was assessed 24 h after the CLP. Graphically, each point represents an animal. The range of 1–3 corresponds to mild sepsis, from 4 to 7 to moderate sepsis and 8 to 11 to severe sepsis. The animals were treated with bosutinib 3 mg/kg 30 min before and 6 h after CLP. (C–F) Inflammatory mediators were quantified by ELISA using plasma or peritoneal lavage collected 24 h after CLP. (C) and (E) exhibit levels of TNF‐α in plasma and peritoneal lavage, respectively, while (D) and (F) exhibit CXCL1 levels in plasma and peritoneal lavage, respectively. Data are presented as mean ± SEM. Statistical analysis: One‐way ANOVA followed by Newman–Keuls post‐test for Figure 2B–F and Mantel‐Cox log‐rank test for Figure 2A. *p* < .05. Bos—bosutinib. *Sham + Vehicle versus CLP + Vehicle; ^#^CLP + Vehicle versus CLP + Bos (3 mg /kg). (A) 10–11 animals per group, representative of three independent experiments. (B) 15–28 animals per group, result of three experiments. (C) Four to five animals per group, representative of two independent experiments. (D, E) Five to six animals per group, single experiment. (F) Five to eight animals per group, single experiment.

To investigate the role of bosutinib on survival, we treated mice with bosutinib (3 mg/kg) starting 30 min prior to the induction of sepsis by CLP. Bosutinib promoted a survival rate of 80% 7 days after sepsis induction (Figure 2A), while untreated septic mice had a survival rate of 45% after the same interval. The sham animals showed a survival rate of 100% at the end of the experiment.

Additionally, we found that septic mice treated with bosutinib had a lower severity score than untreated septic mice, restricting themselves to moderate and mild sepsis (Figure 2B). Sham animals did not present any clinical alteration. The CLP group had two animals with mild, 16 with moderate and nine with severe sepsis. The CLP group treated with bosutinib had 15 animals with mild, 13 with moderate and none with severe sepsis.

### 3.2 Bosutinib reduces plasma and peritoneal levels of TNF‐α and CXCL1 in septic mice

Next, we studied TNF‐α and CXCL1 levels in mice with CLP‐induced sepsis and how bosutinib treatment influences those levels in the plasma and peritoneum of septic mice. There was an increase in the levels of TNF‐α and CXCL1 in plasma and peritoneal lavage of septic animals compared to sham animals (Figure 2C–F). In turn, septic animals treated with bosutinib showed reduced levels of TNF‐α and CXCL1 in both groups.

### 3.3 Bosutinib reduces cellular infiltration and the number of colony‐forming units in peritoneal lavage

**FIGURE 3:**
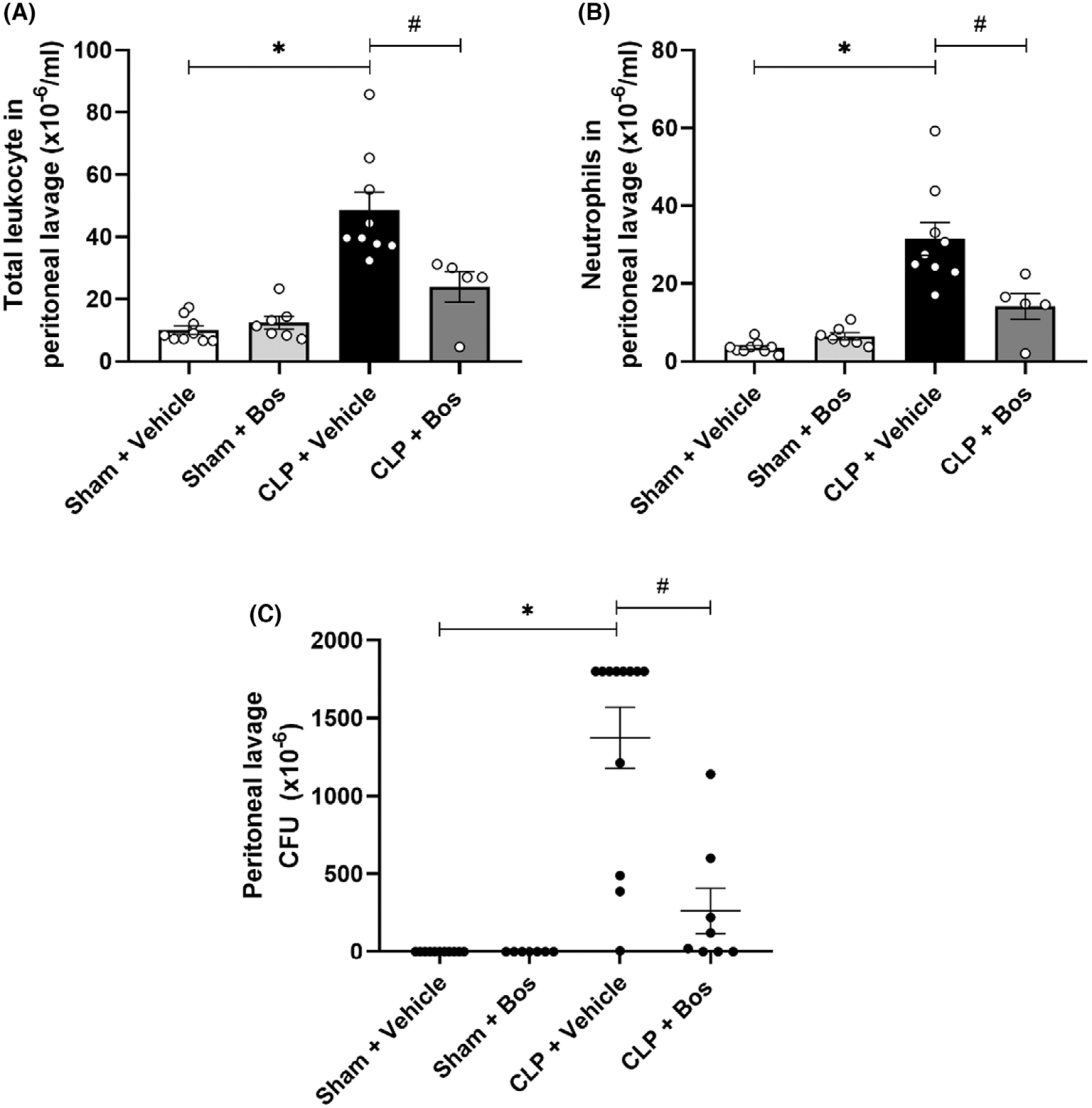
Bosutinib decreases cellular infiltration and bacterial proliferation in peritoneal lavage (PL) in the CLP model. (A) Total cell counts in PL (10^−6^/mL). (B) Differential count of neutrophils. (C) CFU count in PL. Swiss septic mice had cell counts in peritoneal lavage evaluated 24 h after CLP, and colony‐forming units were counted. Sham mice were used as controls. Animals were treated with bosutinib (Bos) 3 mg/kg or Vehicle 30 min before and 6 h after CLP. *Sham + Vehicle versus CLP + Vehicle; ^#^CLP + Vehicle versus CLP + Bos (3 mg/kg). (A, B) Five to nine animals per group, representative of two independent experiments. (C) Seven to 12 animals per group, data from a single experiment.

To study the role of bosutinib on cellular infiltration at the site of infection, we collected peritoneal lavage from the animals and performed a count of leukocytes present in the samples. Septic animals showed a significant increase in total and differential cell counts in peritoneal lavage. Bosutinib treatment reduced the number of total cells in the peritoneal lavage of septic mice and the number of polymorphonuclear cells (Figure 3A,B). Furthermore, to investigate the effect of bosutinib on bacterial proliferation, we cultured the peritoneal lavage for 24 h. In this assay, we obtained pronounced bacterial growth in the CLP group, indicated by the high number of colony‐forming units after 24 h of incubation, compared to the sham group, where we did not find any bacterial growth. The CLP group treated with bosutinib showed a strongly attenuated increase in colony‐forming units compared to vehicle‐treated septic mice, demonstrating that bosutinib impaired bacterial growth (Figure 3C).

### 3.4 Bosutinib reduced leukocyte‐endothelium interactions in the cerebral microvasculature in vivo

**FIGURE 4:**
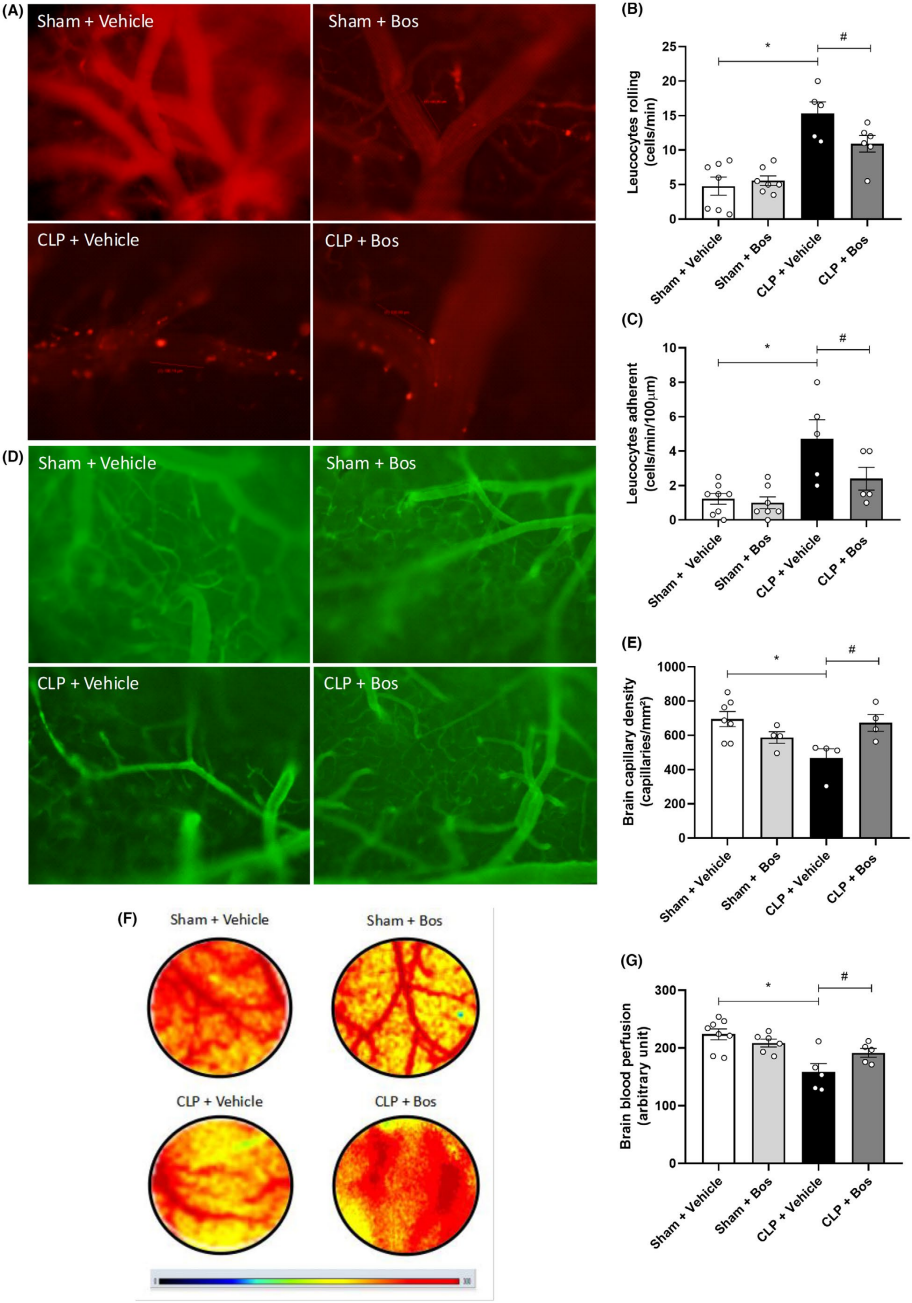
(A–C) Bosutinib diminished the leukocyte‐endothelium interaction in the brain of mice with CLP‐induced sepsis. (A) Images of cerebral microcirculation acquired by fluorescent intravital microscopy. Bos—bosutinib. Bar = 100 μm. 200× magnification. Evaluation of the cerebral endothelium's leukocyte bearing (B) and adhesion (C). (D, E) Bosutinib treatment promotes the maintenance of capillary density and blood perfusion in the cerebral microcirculation of mice with sepsis. The analysis of cerebral capillary density is displayed in images of cerebral microvasculature acquired by intravital microscopy (D) and graphically showing the number of capillaries per mm^**2**^ (E). Bar = 50 μm. 100× magnification. The analysis of cerebral blood perfusion is shown through images of the perfusion of vessels obtained by contrast spectra (laser speckle) (F), and these same data are presented graphically (G). Red represents areas (vessels) with greater blood perfusion. Swiss septic mice treated or not with bosutinib had their functional capillaries, cerebral blood perfusion, and the leukocyte‐endothelium interaction in the post‐capillary cerebral venules assessed 24 h after CLP. The animals were treated with bosutinib 3 mg/kg 30 min before and 6 h after CLP. Data are presented as mean ± SEM. Statistical analysis considered *p* < .05. Bos—bosutinib. *Sham + Vehicle versus CLP + Vehicle; ^#^CLP + Vehicle versus CLP + Bos (3 mg/kg). The number of animals per group varies from four to eight from two independent experiments.

Next, we tested how induction of sepsis affects leukocyte recruitment in cerebral microvessels in vivo using intravital imaging. We found that leukocyte rolling and adhesion increased in septic mice when compared to the sham group. Septic mice treated with bosutinib showed fewer leukocyte rolling and adhesion when compared to vehicle‐treated septic mice, demonstrating that sepsis‐induced leukocyte recruitment is reduced in the presence of bosutinib (Figure 4A–C).

### 3.5 Bosutinib increases the number of functional capillaries and blood perfusion in the brain of septic mice

To investigate the role of bosutinib on cerebral microcirculation, we evaluated cerebral blood perfusion and capillary density in the brain of these animals. Figure 4D,E show that the functional capillary density decreased in septic mice compared to the sham group. Bosutinib treatment increased the number of spontaneously perfused capillaries compared to the untreated CLP group, reversing capillary rarefaction. Figure 4D illustrates photomicrographs of the brain capillaries. In agreement with the previous result, we found that the CLP group had lower cerebral blood perfusion when compared to the sham group, while treatment with bosutinib improved cerebral blood perfusion in septic mice compared to the untreated CLP group (Figure 4F,G). Figure 4F illustrates the photomicrograph of cerebral blood perfusion from the explored cranial window.

### 3.6 Bosutinib decreased levels of pro‐inflammatory cytokines, chemokines, and VEGF in the brain tissue of septic mice

**FIGURE 5:**
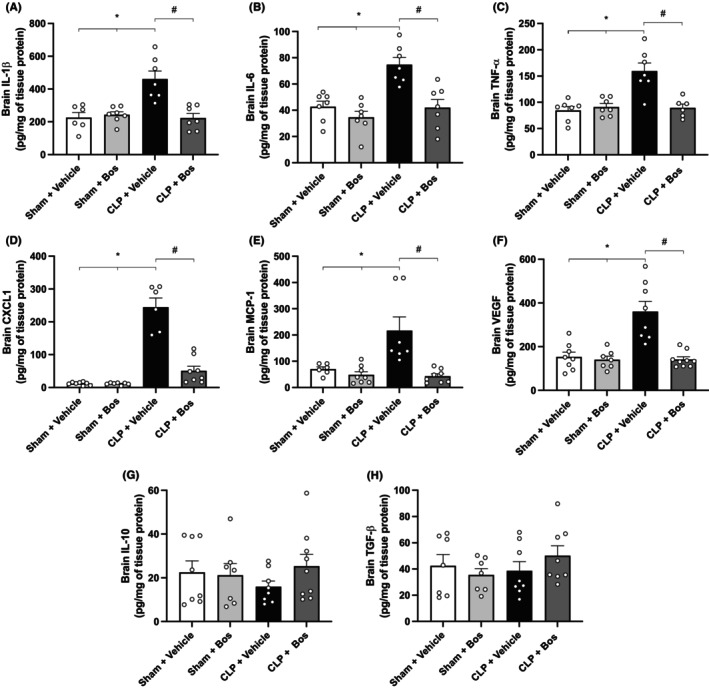
Bosutinib treatment reduced the levels of pro‐inflammatory cytokines, chemokines and VEGF in the brain tissue of septic mice but did not alter the levels of anti‐inflammatory cytokines. Swiss mice underwent cecal ligation and puncture (CLP), and sham animals were used as controls. Data are presented as mean ± SEM. Inflammatory mediators were quantified by ELISA using brain tissue collected 24 h after CLP. (A–H) Displays brain levels of IL1‐β (A), IL‐6 (B), TNF‐α (C), CXCL1 (D), MCP‐1 (E), VEGF (F), IL‐10 (G) and TGF‐β (H). Some animals were treated with bosutinib 3 mg/kg 30 min before and 6 h after CLP. BOS—bosutinib. *Sham + Vehicle versus CLP + Vehicle; ^#^CLP + Vehicle versus CLP + Bos (3 mg /kg). The number of animals per group varies from six to eight.

Next, we studied local inflammatory activity in the brain tissue of mice with CLP‐induced sepsis. To do so, we assessed pro‐inflammatory cytokines IL‐1β, IL‐6, TNF‐α (Figure 5A–C, respectively), chemokines CXCL1 and MCP‐1 (Figure 5D,E, respectively), VEGF (Figure 5F) and anti‐inflammatory cytokines IL‐10 and TGF‐β (Figure 5G,H, respectively) in brain extract of septic mice. Compared to the sham group, we found increased levels of cytokines, chemokines and VEGF in the brain tissues of all septic mice. Interestingly, the CLP group treated with bosutinib reduced the levels of these inflammatory mediators compared to the vehicle‐treated CLP group. We did not detect any difference in the levels of TGF‐β and IL‐10 cytokines in brain tissue between groups.

### 3.7 Bosutinib reduces diffuse alveolar damage caused by sepsis

**FIGURE 6:**
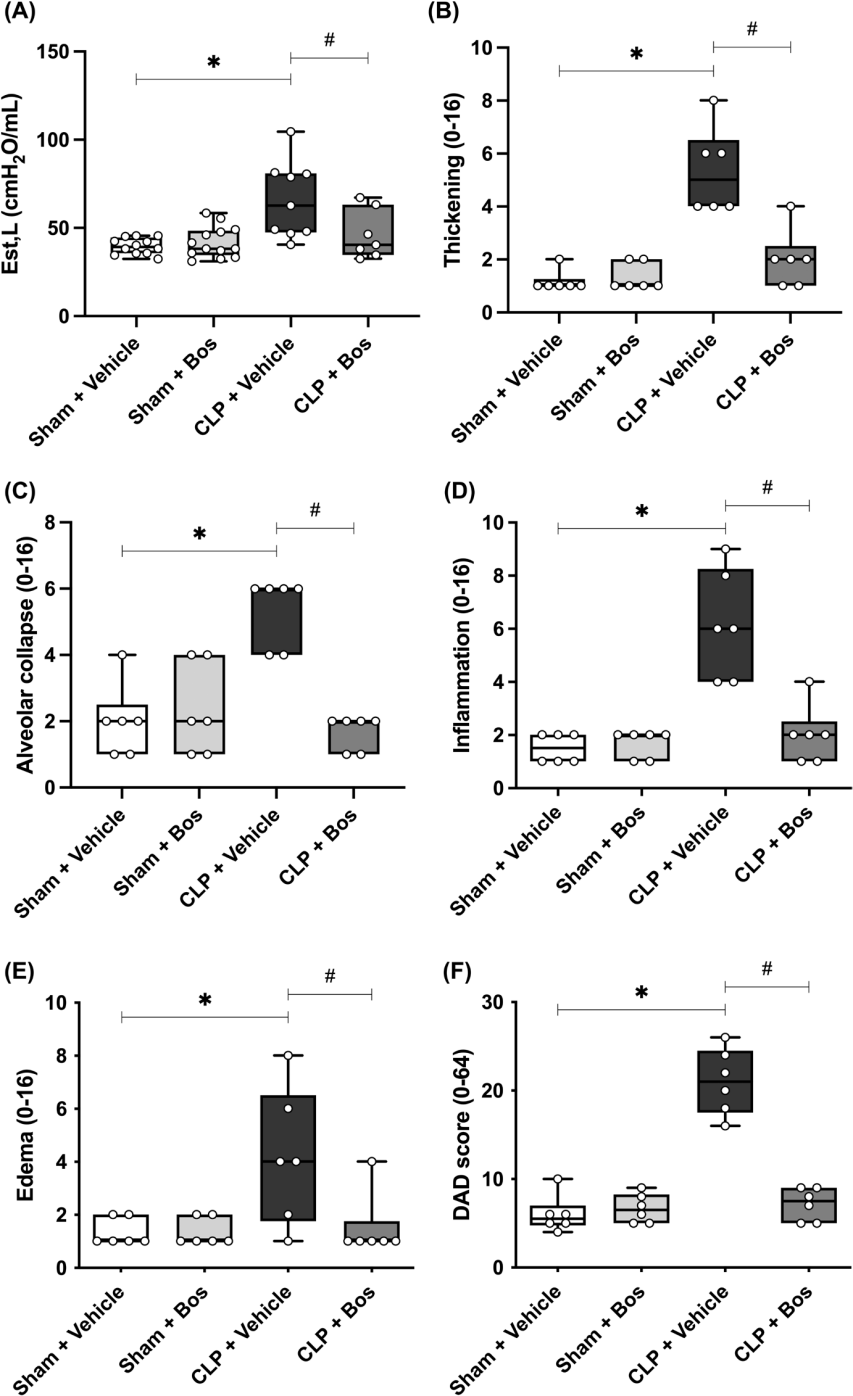
Bosutinib reduces diffuse alveolar damage in septic animals. A scale of 0–4 was used to represent the severity of septal thickening, alveolar collapse, inflammatory infiltration and edema, with 0 standing for no effect and 4 for maximum severity. Additionally, the extent of each score characteristic per field of view was graded on a scale of 0–4, with 0 standing for no appearance and 4 for complete involvement. Scores were calculated as the product of the severity and extent of each feature, ranging from 0 to 16. The cumulated DAD score was calculated as the sum of single score characteristics, yielding a final score ranging from 0 to 64. The animals were treated with bosutinib (Bos) 3 mg/kg 30 min before and 6 h after CLP. Values are medians (interquartile range. *Sham + Vehicle versus CLP; ^#^CLP versus CLP + Bos (3 mg/kg).

Next, we studied lung function in mice with CLP‐induced sepsis. Lung elastance (Est,L) was higher in vehicle‐treated CLP animals than in control animals. Bosutinib treatment reduced Est,L in septic animals compared to the untreated group (Figure 6A). Furthermore, sepsis‐induced animals treated with the vehicle exhibited increased septal thickening, alveolar collapse, edema and cellular infiltration (inflammation) compared to the control group; bosutinib treatment reduced this increased parameter (Figure 6B–D). Diffuse alveolar damage (DAD score) was greater in the CLP group and decreased with treatment (Figure 6F).

## 4 DISCUSSION

Sepsis is a serious public health problem with a high mortality rate (~25%), morbidity and difficult management.^26^, ^27^ Potential therapies have failed to reduce mortality in clinical trials, and no specific drug has yet been approved for sepsis treatment.^21^ Furthermore, bacterial antibiotic resistance is increasing, which makes the scenario more alarming.^28^ Because organ failure as a result of hyperinflammation is the most common cause of death during the first days of sepsis,^29^ therapies aimed at reversion of exaggerated inflammation with the maintenance of immune response to infection are important strategies.^30^ As an important therapeutic target, SFK regulates various physiological processes, including signalling pathways promoting inflammation.^11^, ^13^ Bosutinib is a second‐generation SFK inhibitor that affects SFK and its relevant immunomodulatory potential.^18^, ^19^ In antineoplastic therapies, the daily dose of 400 mg bosutinib provides significant safety,^31^ with no adverse vascular events.^32^ In the present study, we showed that bosutinib at a dose of 3 mg/kg promoted increased survival and reduced severity of sepsis. Of note, the IC50 for inhibition of all SFKs by bosutinib is less than 10 nM.^33^ Previously, we evidenced that dasatinib in an optimized dose (1 mg/kg) showed improved clinical conditions, increased survival and reduced organ damage accompanied by decreased leukocyte recruitment and inflammatory response in mice submitted to CLP.^16^ In the present work, we analysed the effect of bosutinib on systemic inflammation, neuroinflammation and lung function, which has not been addressed previously.

Brain injury occurs in the initial phase of sepsis and favours the progression of disease severity and increased mortality.^34^ The recruitment and activation of circulating leukocytes in microcirculation is a determining event for vascular lesions in the brains of septic animals.^35^ Different studies have indicated an increase in 24 h of leukocyte–endothelium interaction and neutrophil influx in the brain of septic mice.^36^, ^37^ Here, we verified this increase in CLP mice. Bosutinib‐treated septic mice presented a reduction in leukocyte rolling and adhesion in the cerebral microvasculature. In endothelial cells, inhibition of SFK reduces phosphorylation of VE‐cadherin triggered by ICAM‐1 signalling, indicating that efficient neutrophil transmigration requires SFK activity.^38^ Previously, we demonstrated reduced neutrophil recruitment in TNF‐α stimulated cremaster muscle venules of dasatinib‐treated mice.^16^ In another report, it was demonstrated that SFK triple knockout mice (Hck^−/−^Fgr^−/−^Lyn^−/−^) also exhibited reduced adhesion and extravasation of neutrophils in postcapillary venules of the cremaster muscle after challenge with TNF‐α. These results strengthen the idea of using SFK inhibitors to modulate inflammatory diseases with unwanted neutrophil recruitment.^39^

In addition to the pathogenic hyperinflammatory response, sepsis is characterized by establishing immunosuppression, impairing immune responses against the pathogen and resolution of infection.^40^ Thus, administering drugs that markedly inhibiting immune responses can abruptly worsen the septic condition.^20^

Here, we show that bosutinib reduced the number of leukocytes in the peritoneal cavity, preventing exacerbated inflammation and tissue damage. It agrees with a recent publication on selective SYK inhibitor treatment 1 h after the CLP, decreasing inflammation and preventing organ dysfunction.^41^ At the same time, the reduced number of recruited leukocytes in bosutinib‐treated mice can still control local bacterial growth and impair bacterial dissemination. These findings agree with recent publications using animal models and aiming to reduce but not ablate innate immune responses during infection.^16^, ^42^, ^43^

As undisturbed blood flow through the microvasculature is essential for providing oxygen and metabolic substrates to tissues, including the brain,^44^ Awe assessed cerebral blood perfusion in septic mice and found a significant reduction in cerebral perfusion, which has also been reported in earlier studies.^45^ Interestingly, phosphorylation of Src kinase‐mediated NMDA receptors is an important pathophysiological mechanism of neuronal death after an ischemic insult.^46^ SFK inhibitors reduce neurotoxicity in ischemia–reperfusion lesions through several mechanisms, including controlling the regulation of the release of inflammatory mediators.^47^ Moreover, inhibition of Src improves cerebral perfusion by a mechanism dependent on preventing increased VEGF‐induced endothelial permeability, protecting the brain from hypoxia‐associated damage after ischemia induction.^48^ Tissue perfusion is determined by capillary density and blood flow,^49^ and we showed that bosutinib treatment promotes an increase in the number of functional capillaries and improved blood perfusion in the brain of septic mice.

The early production of pro‐inflammatory cytokines and chemokines during neuroinflammation triggered by sepsis provides profound structural and functional changes.^50^ Exaggerated inflammation can cause irreversible damage to nervous tissue, especially vulnerable due to a lack of regenerative and proliferative potential, causing CNS dysfunction.^50^ Notably, the marked release of inflammatory mediators is also related to hemodynamic changes in the cerebral microcirculation.^51^ TLR‐dependent Src phosphorylation is connected to pathways that culminate in the nuclear translocation of CREB, AP‐1 and NF‐kB and transcription of numerous key inflammation genes, including cytokines.^11^, ^13^ We showed that in the brain tissue of septic mice, bosutinib promoted the reduction of the main pro‐inflammatory cytokines, chemokines and VEGF, whose abnormal levels are associated with deleterious effects and progression of the severity of CNS lesions.^52^, ^53^, ^54^ Interestingly, there was no difference between the experimental groups regarding IL‐10 and TGF‐β levels, which may indicate that bosutinib exerts anti‐inflammatory action on the brain by a mechanism that is not dependent on these anti‐inflammatory factors. Systemically, high concentrations of TNF‐α during sepsis contribute to altered chemotaxis, vascular integrity and coagulation, resulting in organ dysfunction.^21^ Furthermore, CXCL1 acts on neutrophil recruitment and release of proteases and ROS in inflamed tissue during infections, amplifying the inflammatory response and damage.^55^ We also found a reduction in TNF‐α and CXCL1 levels in the plasma and peritoneum of bosutinib‐treated mice with sepsis. Herein, we reinforce the ability of bosutinib to control inflammation on a local (as in case of neuroinflammation) as well as a systemic level.

Besides affecting the brain, sepsis is also one of the main causes of acute respiratory distress syndrome (ARDS)^56^ and increases susceptibility to lung injuries induced by mechanical ventilation, widely used in managing the disease.^57^ The exudative phase of ARDS is the initial onset of lung injury, characterized by disruption of the alveolar endothelial barrier with increased permeability and accumulation of edematous fluid.^58^ ARDS caused by pulmonary or extrapulmonary insults increases lung elastance.^22^ In a previous study using a CLP model, an increase in lung elastance, alveolar thickening, alveolar collapse and neutrophil infiltration in septic mice was observed.^59^ Our results show that sepsis‐induced animals significantly increased alveolar elastance, alveolar thickening, alveolar collapse, inflammation and diffuse alveolar damage (DAD) score. However, treatment with bosutinib reduced all these parameters, demonstrating a possible protective role in sepsis‐induced pulmonary damage.

Some limitations of our study need to be addressed in further studies. First, bosutinib treatment was started before sepsis was induced. We chose this treatment protocol as this was the first time bosutinib was investigated in a sepsis model. Using bosutinib before the onset of sepsis provided the best approach to test whether bosutinib has any beneficial effect during sepsis. The other limitation is that we do not use female mice in the experiments. We justify that because sepsis incidence and severity occur in male patients.^60^ In addition, male animals also appear to respond in a more amplified manner to inflammatory stimuli.^61^ Female animals are also subject to higher oestrogen levels, which are linked to inhibiting inflammatory pathways.^62^ Additional studies need to be performed with female animals and bosutinib treatment starting after sepsis induction to establish a potential role of bosutinib as adjuvant therapy.

## 5 CONCLUSION

We investigated the potential protective role of Bosutinib treatment in mice with CLP‐induced sepsis and found interesting immunomodulatory responses by Bosutinib. This consisted in reducing the inflammatory activity in plasma, peritoneal lavage and brain tissue of septic mice. These immunomodulatory effects contributed to reducing the number of cells in the peritoneal lavage of septic animals, including a reduction in neutrophils, while also reducing the number of colony‐forming units. Furthermore, bosutinib improved key lung function parameters involved in the pathology of ARDS. As a potent SFKI, bosutinib can regulate the cellular responses of leukocytes, reducing the release of cytokines and chemokines and decreasing inflammation, recruitment and transmigration of immune cells, modulating exacerbated inflammation and its well‐known damaging role during sepsis.

Furthermore, bosutinib treatment reduced leukocyte rolling and adhesion in the cerebral microcirculation of septic mice with improved brain capillary density and blood perfusion. Finally, bosutinib treatment reduced sepsis severity and overall mortality. Thus, bosutinib, a potent SFKI, beneficially modulates inflammatory responses in septic mice, reinforcing interest in future research, including clinical trials to test bosutinib as an option in patients with severe infectious diseases, including sepsis.

## AUTHOR CONTRIBUTIONS

Conceptualization, methodology, research, data curation: Cunha, C. M. C.; Abreu, V. H. P.; Estato, V.; Moraes, B. P. T.; Soraes, G. M. V.; Oliveira, G. P.; Silva, J. D.; Silva, P. L.; Immler, R.; Rocco, P. R.; Gonçalves‐de‐Albuquerque, C. F.; Silva, A. R.; Sperandio, M. Project management: Gonçalves‐de‐Albuquerque, C. F.; Silva, A. R.; Castro‐Faria‐Neto, H. C.; Sperandio, M. Financing acquisition: Bozza, P. T.; Gonçalves‐de‐Albuquerque, C. F.; Silva, A. R.; Castro‐Faria‐Neto, H. C. Written: Cunha, C. M. C.; Abreu, V. H. P.; Gonçalves‐de‐Albuquerque, C. F. All authors read and agreed with the published version of the manuscript.

## CONFLICT OF INTEREST STATEMENT

The authors declare no conflict of interest.

## Data Availability

The data that support the findings of this study are available from the corresponding author.
